# Inclusion body myositis in a patient with chronic myeloid leukemia treated with dasatinib: a case report

**DOI:** 10.1186/s13256-015-0674-9

**Published:** 2015-09-16

**Authors:** Naif I. AlJohani, Simon Carette, Jeffrey H. Lipton

**Affiliations:** 1 Seattle Cancer Care Alliance, University of Washington, 337 NE 103rd Street, Apartment # 448, Seattle, WA 98125 USA; 2 Division of Rheumatology, University Health Network, Mount Sinai Hospital, Toronto, Canada; 3 Department of Hematology/Medical Oncology, Princess Margaret Cancer Centre, Toronto, Canada

**Keywords:** Chronic myelogenous leukemia (CML), creatine kinase (CK), dasatinib, inclusion body myositis, inflammatory myopathy, non-Hodgkin’s lymphoma, progressive muscle weakness, tyrosine kinase inhibitor (TKI)

## Abstract

**Introduction:**

Chronic myelogenous leukemia is often treated using tyrosine kinase inhibitors such as dasatinib. Here we describe a rare case of inflammatory myopathy in a patient with chronic myelogenous leukemia treated with the tyrosine kinase inhibitor dasatinib.

**Case presentation:**

A 69-year-old Caucasian man with imatinib-resistant chronic myelogenous leukemia achieved complete molecular remission in response to dasatinib therapy. However, from a normal initial serum creatine kinase level, he developed elevated serum creatine kinase levels and gradual-onset progressive muscle weakness after dasatinib therapy was initiated. Our patient was eventually diagnosed with inclusion body myositis. However, we were unable to determine the mechanism underlying the dasatinib-associated muscle weakness. Given the efficacy of dasatinib in the treatment of chronic myelogenous leukemia and our patient’s mild symptoms of inclusion body myositis, he continued to receive dasatinib under close clinical and laboratory observation.

**Conclusion:**

Despite the wide use of dasatinib and its documented safety, we report a case of severe muscle injury of unknown etiology. Therefore, patients with chronic myelogenous leukemia receiving dasatinib and perhaps all tyrosine kinase inhibitors should be carefully monitored for signs of muscle injury, especially if this is associated with significant elevations in serum creatine kinase levels.

## Introduction

Chronic myelogenous leukemia (CML) accounts for approximately 15–20% of all leukemia cases in adults in the USA [1]. Dasatinib is a potent oral multi-Bcr/Abl and Src family tyrosine kinase inhibitor (TKI) that also inhibits platelet-derived growth factor receptor (PDGFR) and c-Kit activity [2, 3]. Dasatinib was approved by the US Food and Drug Administration as a first-line therapeutic for use in patients with CML [4] and Philadelphia chromosome-positive acute lymphoblastic leukemia [5].

The common side effects of dasatinib therapy include recurrent pleural effusions, myelosuppression, and rash. Other treatment-related adverse events include mild to moderate diarrhea, peripheral edema, and headache [6, 7]. Elevated levels of serum creatine kinase (CK) have been reported to occur in <1% of patients [8]. In a pivotal clinical study comparing imatinib with dasatinib in patients with newly diagnosed chronic phase CML, serum CK level elevation was reported in only one out of 159 patients [9]. In another study, elevated serum CK levels were reported in a patient with dasatinib-associated ventricular arrhythmia [10]. Severe reproducible rhabdomyolysis has been reported in a patient with CML receiving imatinib; however, the occurrence of inclusion body myositis (IBM) and/or rhabdomyolysis in patients receiving dasatinib has not been previously reported [11]. This is to the best of our knowledge the first report of a patient with imatinib-resistant CML who achieved complete molecular remission in response to second-line dasatinib therapy, but who subsequently had significantly elevated serum CK levels and was diagnosed with IBM.

## Case presentation

**Fig. 1:**
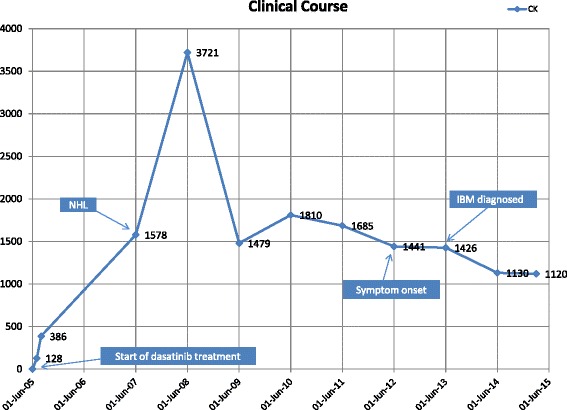
Clinical course. *IBM* inclusion body myositis, *NHL* non-Hodgkin’s lymphoma

A 69-year-old Caucasian man who was diagnosed with chronic phase CML in 1990 demonstrated a reasonable response to initial treatment with interferon and omacetaxine mepesuccinate (homoharringtonine), which was discontinued 14 years later when he began receiving imatinib mesylate (Gleevec) therapy. Our patient became resistant to imatinib one year after its first administration and developed accelerated phase disease; dasatinib was prescribed as second-line treatment. His initial serum CK level was normal. A marked elevation in his serum CK level was noted (range 1,000–4,000 units/L; Fig. 1) immediately after the initiation of dasatinib therapy. During the subsequent 7 years, our patient did not show any evidence of musculoskeletal symptoms but did develop edema and several pleural effusions, both known complications of dasatinib therapy. Two years after starting dasatinib treatment, our patient presented with enlarged neck nodes consequent to non-Hodgkin’s lymphoma. He underwent chemoradiation therapy with three cycles of rituximab and cyclophosphamide, doxorubicin, vincristine, and prednisone (CHOP) combined with involved-field radiation therapy, and he achieved remission.

Our patient was not receiving any drugs associated with muscle injury, but 5 years after developing non-Hodgkin’s lymphoma, gradual-onset proximal muscle weakness was noted in his legs. Our patient reported difficulties in standing from a seated position and in climbing stairs. On physical examination, his eye movements were full but bilateral weakness of his orbicularis oculi was evident. Weakness was minimal in his upper limbs and restricted to his long finger flexors and his flexor digitorum profundus 4 and 5. In his lower limbs, there was considerable wasting of his quadriceps, but sparing of his rectus femoris.

A muscle biopsy of our patient’s right vastus showed significant fibrosis, inflammation, and adipose tissue replacement of the interstitium. Inflammatory cell infiltrate was apparent in the perimysium, concentrated in the endomysium and necrotic fibers, and present to a lesser extent in his normal muscle fibers. The sampled vasculature appeared normal without evidence of vasculitis. The clinical and histological findings were compatible with a diagnosis of an inflammatory myopathy that was most likely IBM.

Because the dasatinib was efficacious in the maintenance of complete molecular remission of our patient’s CML, the drug was not discontinued. In addition, the reported symptoms were mild and unlikely to respond to corticosteroids, so our patient did not receive any additional treatment, but was placed under close observation for symptom progression. At the time of writing, his CK levels remain unchanged, his clinical status has not worsened, and he remains with levels of bcr-abl indicating a minimum of molecular response 4.5. He recently developed shingles that was treated with famciclovir and pregabalin.

In the case reported here, although dasatinib resulted in complete molecular remission of CML, our patient developed elevated serum CK levels and muscle weakness. To date, elevated serum CK levels have been rarely reported in patients undergoing dasatinib therapy, whereas IBM and inflammatory myopathy have never been reported. However, myalgia and muscle spasms have been reported as adverse reactions in approximately 4% of patients with newly diagnosed chronic phase CML receiving dasatinib (Sprycel) and in 7% of those resistant or intolerant to imatinib therapy (minimum follow-up of 36 months) [12]. Other post marketing surveillance studies have reported variable ranges of the incidence of myalgia and muscle spasms [13], although muscle weakness has never been reported as an adverse reaction to dasatinib therapy. The relationship of dasatinib and, for that matter, any TKI therapy with elevated serum CK levels is unknown. Imatinib inhibits both PDGFR and c-ABL, which are expressed by muscle tissue, and inhibition of these enzymes may lead to muscle toxicity, which could increase serum CK levels [14–16]. The rapid increase in serum CK levels in our patient with the start of dasatinib therapy, together with the continued and marked increase throughout the treatment period, might indicate a possible association with dasatinib. Frank IBM associated with any TKI is a rarity and there is no cause and effect proven. In addition, although IBM is associated with advancing age and our patient was 69 years old, its occurrence is known to be rare [17].

## Conclusions

We have reported the case of a patient with CML who developed significantly elevated serum CK levels while receiving dasatinib therapy, and who was subsequently diagnosed with IBM. This is what we believe to be the first report of a patient developing muscle weakness while receiving dasatinib, although causality could not be established. Furthermore, the plausible mechanisms underlying dasatinib-induced muscle injury could not be determined. Although dasatinib has been widely used in clinical practice and is safe, based on our experience, severe muscle injury should be investigated. Patients with CML receiving dasatinib or other TKIs should be carefully monitored for symptoms or signs of muscle injury, especially if this is associated with significant elevations in serum CK levels. Further studies are needed to determine the underlying pathophysiology of muscle injury in such patients.

## Consent

Written informed consent was obtained from the patient for publication of this case report. A copy of the written consent is available for review by the Editor-in-Chief of this journal.
